# KILchip v1.0: A Novel *Plasmodium falciparum* Merozoite Protein Microarray to Facilitate Malaria Vaccine Candidate Prioritization

**DOI:** 10.3389/fimmu.2018.02866

**Published:** 2018-12-11

**Authors:** Gathoni Kamuyu, James Tuju, Rinter Kimathi, Kennedy Mwai, James Mburu, Nelson Kibinge, Marisa Chong Kwan, Sam Hawkings, Reuben Yaa, Emily Chepsat, James M. Njunge, Timothy Chege, Fatuma Guleid, Micha Rosenkranz, Christopher K. Kariuki, Roland Frank, Samson M. Kinyanjui, Linda M. Murungi, Philip Bejon, Anna Färnert, Kevin K. A. Tetteh, James G. Beeson, David J. Conway, Kevin Marsh, Julian C. Rayner, Faith H. A. Osier

**Affiliations:** ^1^KEMRI-Wellcome Trust Research Programme, Centre for Geographic Medicine Research—Coast, Kilifi, Kenya; ^2^Centre for Infectious Diseases, Parasitology, Heidelberg University Hospital, Heidelberg, Germany; ^3^Department of Biochemistry, Pwani University, Kilifi, Kenya; ^4^Arrayjet, Innovative Microarray Solutions, Edinburgh, United Kingdom; ^5^Department of Pathology, University of Cape Town, Cape Town, South Africa; ^6^Department of Tropical and Infectious Diseases, Institute of Primate Research, Nairobi, Kenya; ^7^Cellular and Molecular Immunology, Vrije Universiteit Brussels, Brussels, Belgium; ^8^Centre for Tropical Medicine and Global Health, Nuffield Department of Clinical Medicine, University of Oxford, Oxford, United Kingdom; ^9^Division of Infectious Diseases, Department of Medicine Solna, Karolinska Institutet, Stockholm, Sweden; ^10^Department of Infectious Diseases, Karolinska University Hospital, Stockholm, Sweden; ^11^Immunology and Infection Department, London School of Hygiene and Tropical Medicine, London, United Kingdom; ^12^Burnet Institute, Melbourne, VIC, Australia; ^13^Central Clinical School, Monash University, Melbourne, VIC, Australia; ^14^Department of Medicine, University of Melbourne, Melbourne, VIC, Australia; ^15^Pathogen Molecular Biology Department, London School of Hygiene and Tropical Medicine, London, United Kingdom; ^16^African Academy of Sciences, Nairobi, Kenya; ^17^Wellcome Sanger Institute, Hinxton, Cambridge, United Kingdom

**Keywords:** *Plasmodium falciparum*, merozoite, antibodies, vaccine candidates, protein microarray, bioinformatics

## Abstract

Passive transfer studies in humans clearly demonstrated the protective role of IgG antibodies against malaria. Identifying the precise parasite antigens that mediate immunity is essential for vaccine design, but has proved difficult. Completion of the *Plasmodium falciparum* genome revealed thousands of potential vaccine candidates, but a significant bottleneck remains in their validation and prioritization for further evaluation in clinical trials. Focusing initially on the *Plasmodium falciparum* merozoite proteome, we used peer-reviewed publications, multiple proteomic and bioinformatic approaches, to select and prioritize potential immune targets. We expressed 109 *P. falciparum* recombinant proteins, the majority of which were obtained using a mammalian expression system that has been shown to produce biologically functional extracellular proteins, and used them to create KILchip v1.0: a novel protein microarray to facilitate high-throughput multiplexed antibody detection from individual samples.

The microarray assay was highly specific; antibodies against *P. falciparum* proteins were detected exclusively in sera from malaria-exposed but not malaria-naïve individuals. The intensity of antibody reactivity varied as expected from strong to weak across well-studied antigens such as AMA1 and RH5 (Kruskal–Wallis H test for trend: *p* < 0.0001). The inter-assay and intra-assay variability was minimal, with reproducible results obtained in re-assays using the same chip over a duration of 3 months. Antibodies quantified using the multiplexed format in KILchip v1.0 were highly correlated with those measured in the gold-standard monoplex ELISA [median (range) Spearman's R of 0.84 (0.65–0.95)]. KILchip v1.0 is a robust, scalable and adaptable protein microarray that has broad applicability to studies of naturally acquired immunity against malaria by providing a standardized tool for the detection of antibody correlates of protection. It will facilitate rapid high-throughput validation and prioritization of potential *Plasmodium falciparum* merozoite-stage antigens paving the way for urgently needed clinical trials for the next generation of malaria vaccines.

## Introduction

Protein microarrays are increasingly used in the “omic” era of research in multiple formats that share the basic requirement to investigate interactions of tens to thousands of proteins simultaneously (1). They have had important translational applications in biomarker discovery to guide patient diagnosis, treatment and prognosis, as well as in drug discovery and vaccine antigen identification (2). Protein microarrays have facilitated a rapid, systematic and high-throughput approach to probing an entire pathogens' proteome or fraction thereof for immunoreactivity, in an approach that forms part of a reverse vaccinology workflow. These have aided in the discovery of potential diagnostic markers for *Mycobacterium tuberculosis* and *SARS-coronavirus* as well as potential vaccine candidates in over 30 human pathogens including *Plasmodium falciparum* (2, 3).

*P. falciparum* malaria causes ~450,000 deaths per year (4), and is of major public health importance to sub-Saharan Africa (5). Recent gains in reducing the burden appear to have stalled despite ongoing control efforts (4, 6). Efforts to design a highly effective vaccine that would protect against this disease have been hampered by the complexity of the organism and its' multi-stage life cycle: its genome encodes >5,300 proteins that are expressed variably in different tissues as the infection develops in the host (7). Coupled to this is an impressive array of strategies for generating protein polymorphisms or protein variants and redundant erythrocyte invasion pathways, which facilitate immune evasion (8–10). Consequently, although efforts to develop a highly effective malaria vaccine have been on-going for over a century, this goal has yet to be achieved. The current leading vaccine candidate against *P. falciparum* malaria has limited efficacy and induces only short-lived protective immunity (11, 12).

Multiple *P. falciparum* and/or *P. vivax* protein arrays have been designed over the past decade to help identify and prioritize potential malaria vaccine antigen candidates. The majority of these arrays have been manufactured using either the *E. coli*-based or the wheat germ cell free *in-vitro* transcription/translation expression system, with the largest to date including ~30% of the entire *P. falciparum* proteome (13–17). Protein selection was based on stage-specific transcription or protein expression, sub-cellular localization, secondary protein structures or documented immunogenicity in human and animal models. However, the *in-vitro* transcription/translation systems are relatively poor at generating functional surface proteins, which frequently require disulphide bonding and/or post-translational modification to attain their correct three-dimensional structure. Nevertheless, subsequent studies have down-selected proteins from this initial panel (18–31), indicating that essentially >75% of the parasite genome has yet to be evaluated in the context of immunity. A few additional proteins have been tested independently in smaller scale studies accounting for only a marginal increase in the proportion of the parasite proteome evaluated to date (32–34). These studies have rationally selected merozoite proteins that were established or plausible targets of antibodies, and evaluated antibody associations with protection in longitudinal studies using standard ELISA-based approaches (32, 33). They highlighted the importance of evaluating a broad repertoire of antigens and combinations of antibody responses in studies of acquired immunity. However, there still remains a need for a common platform with standardized protein expression and high-throughput antibody detection methods that can be applied widely across different clinical studies (35). This would accelerate identification of protective antibody targets and facilitate the comparisons between studies and populations.

To contribute to vaccine candidate discovery, as well as the validation and prioritization of existing candidates for clinical trials, we designed a novel protein microarray. We focused on the merozoite stage that is a target of immunity that can prevent or reduce the clinical symptoms of malaria. As per the case with other infectious diseases (36, 37), we hypothesized that proteins on or associated with the surface of the invasive *P. falciparum* merozoite would be accessible targets for protective antibodies (33). We mined the literature to identify multiple potential surface-associated merozoite proteins (32–34, 38–43) and added new proteins that were identified as immunogenic in adults from malaria-endemic countries and had proteomic and/or bioinformatic features suggestive of merozoite surface-localization, secretion and/or involvement in erythrocyte invasion (44). We expressed and purified these proteins and printed them on a custom microarray, which we refer to as KILchip v1.0 for its origin at the KEMRI-Wellcome Trust Research Programme in Kilifi, Kenya where the majority of the work was carried out. We demonstrate that KILchip v1.0 is highly specific, has minimal inter- and intra-assay variation, and is strongly correlated with equivalent data acquired using the gold-standard monoplex ELISA.

## Materials and Methods

### Protein Selection

We aimed to design a microarray that would include proteins already considered as vaccine candidates, as well as novel proteins that had not been studied in the context of protective immunity. This would serve multiple purposes: (i) validation of existing vaccine candidates in new sample sets, (ii) identification of novel potential candidates and (iii) facilitation of head-to-head comparisons of all selected candidates in the same experiment. To this end, we selected a panel of antigens previously published in Zenonos et al. (38), Richards et al. (32), Crosnier et al. (39), Tetteh et al. (34), Raj et al. (43), Polley et al. (40, 45), Kimbi et al. (46), Metzger et al. (47), Taylor et al. (41), and Burghaus and Holder (48), for inclusion in the KILchip v1.0. These proteins are known or predicted to be anchored or associated with the surface of merozoites, secreted from its apical organelles or involved in erythrocyte invasion and have been shown to correlate with protection from clinical malaria (32, 33). We also included 28 novel proteins selected through a protein discovery pipeline that employed proteomic approaches for the detection of proteins that were either immunogenic or located on the surface of merozoites (44). Down-selection criteria for the novel proteins included either (i) the presence of a predicted N-terminal signal peptide and/or transmembrane domain(s), (ii) upregulated transcription at the late stages of the asexual life cycle, (iii) a predicted role in merozoite invasion (49) and (iv) novel with regards to a potential role in protective immunity.

### Recombinant *P. falciparum* Protein Expression

Plasmids containing codon-optimized genes of interest were either obtained from the plasmid repository Addgene (https://www.addgene.org) or newly synthesized by GeneartAG as has been previously published (38, 39). Briefly, predicted signal peptides and transmembrane domains were excluded and the serine or threonine amino acid residues in all potential N-linked glycosylation sites (NXS/T) were substituted with alanine. Codon-optimized genes of interest were sub-cloned into a derivative of the pTT3 expression vector (also obtained from Addgene) that contained an N-terminal signal peptide derived from the mouse *kappa* light chain to drive secretion of antigen and a rat Cd4 domains 3 and 4 tag followed by a hexa-histidine tag for protein purification (39). Proteins were subsequently expressed using the Expi293 expression system (Invitrogen) according to manufacturer's instructions. Briefly, Expi293F cells were cultured to a density of 2.0 × 10^6^ cells/ml and transfected with expression vectors using the Expifectamine 293 transfection reagent (Invitrogen). Cells were then incubated at 37°C with 8% CO_2_ in an orbital shaker at 125 rpm. Culture supernatants were harvested 6 days post-transfection and proteins purified using Ni-NTA purification columns (Invitrogen). The majority of proteins included in KILchip v1.0 were expressed using the above mammalian expression system.

A minority of proteins were expressed in *E. coli* using pGEX-2T and pMAL-c2X vectors to produce fusion proteins with the carriers glutathione-S-transferase (GST) and maltose-binding protein (MBP), respectively. These were transformed into BL21 (DE3) *E. coli* cells and expressed as previously described (34, 40–42). The gene encoding *Pf* SEA1 protein was amplified from *P. falciparum* 3D7 cDNA using previously described primers (43). The PCR products were cloned into pEXP5-NT/TOPO expression vector and transformed into BL21 (DE3) pLysS *E.coli* cells. Cell expansion, induction of protein expression and subsequent purification was performed as previously published (34, 40–42). Purified recombinant proteins were dialysed into phosphate buffered saline and quantified using NanoDrop (Thermo scientific) before printing onto nitrocellulose slides. Further details are provided in the Supplementary information.

### LC-MS/MS Analysis, Protein Validation, and Circular Dichroism Spectroscopy

Five to fifteen μg of purified recombinant proteins were denatured in 50 mM Tris-HCL pH 8.0 (Sigma) containing 8 M urea (Sigma). Proteins were reduced with 40 mM dithiothreitol (Sigma), alkylated with 80 mM iodoacetamide (Sigma) and precipitated using cold acetone. Pelleted proteins were resuspended in 15 μl of 6 M urea in 50 mM Tris-HCL pH 8.0 and digested with trypsin/Lys-C mix (Promega) according to manufacturer's instructions using the two step in-solution digestion. Peptides were desalted using P10 c18 pipette ZipTips (Millipore), dried using the Speedvac concentrator (Thermo Scientific) and resuspended in 15 μl of 99% H_2_0, 1% acetonitrile, and 0.1% formic acid. Peptides (5 μl) were loaded using a Dionex Ultimate 3,000 nano-flow ultra-high-pressure liquid chromatography system (Thermo Scientific) on to a 75 μm × 2 cm C18 trap column (Thermo Scientific). Chromatographic separation of peptides was carried out on a reverse-phase 50 cm-long column (Thermo Scientific) maintained at 40°C over a 60-min elution gradient (2–40% of mobile phase B; 80% acetonitrile with 0.1% formic acid) at a flow rate of 0.3 μl/min. Peptides were measured using LC instrumentation consisting of a Dionex Ultimate 3,000 nano-flow ultra-high-pressure liquid chromatography system (Thermo Scientific) coupled via a nano-electrospray ion source (Thermo Scientific) to a Q Exactive Orbitrap mass spectrometer (Thermo Scientific). The ms^1^ settings were: Resolution, 70,000; Automatic gain control (AGC) target, 3e6; maximum injection time, 100 ms; scan range, 380–1600 m/z; while the ms^2^ settings were: Resolution, 17,500; AGC target, 5e4; maximum injection time, 100 ms; isolation window, 1.6 m/z. The top 10 most intense ions were selected for ms^2^ and fragmented with higher-energy collision fragmentation using normalized collision energy of 28 and these ions were subsequently excluded for the next 20 s. Mass spectrometry raw files were searched on Proteome Discoverer software version 1.3.0.339 (Thermo Scientific) using the Mascot server (Matrix Science) using a concatenated database of human and 3D7 *Plasmodium falciparum* protein FASTA sequences. Cysteine carbamidomethylation was set as a fixed modification and deamidation of asparagine or glutamine and methionine oxidation as variable modifications. The false discovery rate (FDR) was set to 0.01 for both proteins and peptides and a maximum of two missed cleavages were allowed in the database search. A minimum of two unique peptides for a protein was considered a positive identification.

Two proteins were selected for circular dichroism spectroscopy analysis (50). Briefly, phosphate buffer (50 mM NaH_2_PO_4_ pH 8.0, 0.1 M NaCl) and samples in phosphate buffer were degassed in a vacuum at room temperature and the CD spectra recorded on a J-715 spectropolarimeter (Jasco). Six accumulations were taken per protein with continuous scans taken using a 1 mm (0.1 cm) quartz cuvette, a scan rate of 50 nm/min, a band width of 1.0 nm and a resolution of 0.5 nm. The raw CD data (ellipticity θ in mDeg) were normalized for protein concentration and the number of residues yielding the mean residue ellipticity [θ] in mDeg·cm2·dmol^−1^·res^−1^.

### Data Availability

The mass spectrometry raw files generated and analyzed in the current study have been deposited to the ProteomeXchange Consortium51 (PXD011746), via the MassIVE partner repository (MSV000083144), under the following title: KILchip v1.0 A novel *Plasmodium falciparum* merozoite protein microarray to facilitate malaria vaccine candidate prioritization. The FTP for the dataset is available here: ftp://massive.ucsd.edu/MSV000083144.

### KILchip v1.0 Protein Microarray Assay

#### Overview

**Figure 1:**
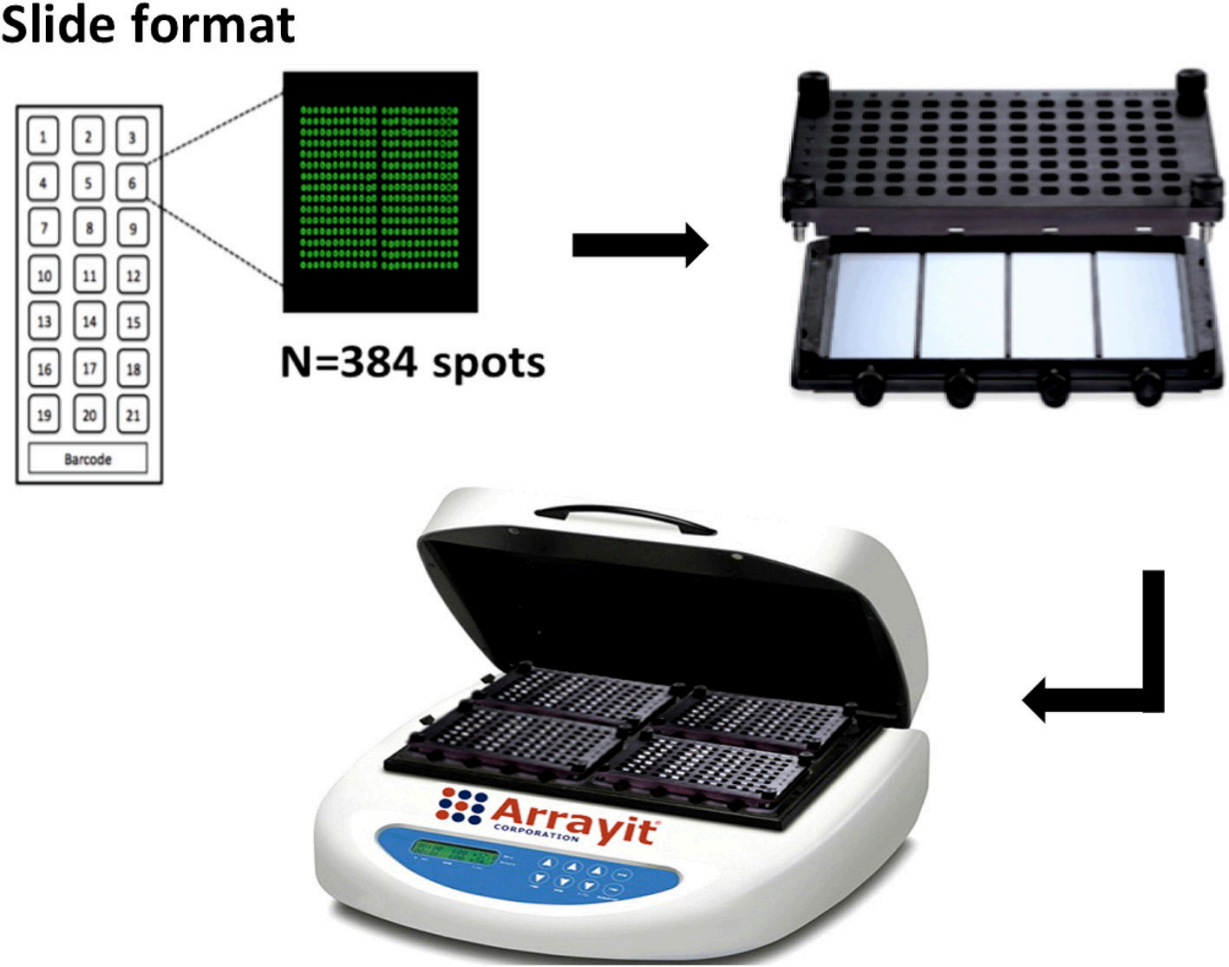
Configuration of KILchip v1.0. Individual slides contain 21 identical mini-arrays comprised of protein spots and a barcode for identification. Each mini-array contains 384 individual spots that include proteins and controls printed in triplicate. Four slides are assembled onto a hybridization cassette, four of which are simultaneously processed in a microarray hybridization work-station (image from Arrayit Corporation, used with permission).

We designed our protein microarray to test 21 unique serum samples per slide with a customized barcode for slide identification. Four slides fitted into a 4 × 24 hybridization cassette (Arrayit Corporation ARYC), thus accommodating 84 samples per cassette and 336 samples per hybridization workstation (ARYC), each of which contains 4 hybridization cassettes (Figure 1).

#### Microarray Protein Map

Each slide contained 21 identical protein mini-arrays. Each mini-array had 384 features (printed spots) at a volume of 400 pl per spot. Recombinant *P. falciparum* proteins (*n* = 111) and controls (*n* = 17) were printed on each mini-array in triplicate, at the same concentration, using the same printer (Ultra Marathon micro-arrayer, ArrayJet) and printing protocol. The first control spots were Alexafluor^647^ human IgG (Jackson ImmunoResearch) that served as landmarks demarcating the four edges of each mini-array (4 spots to match the edges of each mini-array). Purified human IgG (Jackson ImmunoResearch, 1 spot), served as the second control that confirmed the activity of the secondary antibody utilized in the assay. Protein printing buffer (30% glycerol/PBS, 9 spots) was used as the third set of controls that allowed for the monitoring of background reactivity and for the detection of any potential protein carryover during printing. The last sets of controls were the CD4, MBP, and GST proteins (1 spot each) to control for any potential antibody reactivity against the tags, each of which is present in one or more of the recombinant *P. falciparum* antigens. Thus, each mini-array contained a total of 384 features: 333 derived from 111 *P. falciparum* recombinant proteins (109 proteins, 2 of which were included twice) and 51 from 17 controls, all printed in triplicate.

#### Microarray Printing

We optimized the concentrations for individual proteins, serum samples and the secondary antibody by checkerboard titration. Proteins were printed on nitrocellulose slides (ONCYTE SuperNOVA, GraceBio) at a concentration of 250 μg/ml using the Ultra Marathon micro-arrayer (ArrayJet) with the Inkjet printing technology and the command center 1.5.0.1 (ArrayJet). Printing was carried out at 50% relative humidity and at 18°C. As a drying step, slides were incubated overnight at 18°C in the arrayer after printing, before storage in slide boxes with dessicant at 4°C until use. A salt scan was carried out at a high photomultiplier (PMT) at the 532 nm wavelength (green channel) to verify post-printing quality and at the 635 nm wavelength (red channel) to allow visualization of the landmark spots.

#### Antibody Detection

Printed slides were carefully assembled onto the hybridization cassette and sealed using silicone gaskets (ARYC) to form leak-proof individual wells. We modified a published protocol for the detection of antibodies (51). Briefly, wells were washed thrice with 0.1% Tween 20/HEPES buffered saline (1.4 M NaCl, 50 mM KCl, 20 mM CaCl_2_, 10 mM MgCl_2_, 100 mM HEPES; HBS) followed by HBS to remove any unbound proteins. Non-specific binding to the slide surface was prevented by blocking with 200 μl of 2% BSA/0.1% Tween 20/HBS for 2 h at room temperature while rotating on a microarray hybridization station (ARYC) at 350 rpm. Wells were subsequently washed thrice and incubated overnight at 4°C with 150 μl of serum diluted 1:400 and rotating at 350 rpm on the hybridization station. Thereafter, wells were washed as described above and incubated with 150 μl of donkey anti-human IgG-Fcγ fragment specific Alexafluor^647^ for 3 h at room temperature followed by three washes. Slides were carefully disassembled from the hybridization cassettes, rinsed thrice in distilled water and dried by centrifugation at 300 g for 5 min using a combiSlide adapter (Eppendorf) and stored in slide boxes in the dark. Slides were scanned using a Genepix 4,000 B scanner coupled to the GenePix Pro & Microarray Acquisition and Analysis Software (Molecular Devices).

### ELISA

A standard protocol for an indirect ELISA was performed as has been previously described (52–54). Briefly, a pre-determined concentration for each recombinant protein was either heat-treated at 80°C for 10 min or left untreated and coated overnight at 4°C on 96-microwell ELISA plates (Dynex 4HBX Immunolon). Wells were washed four times in PBST (phosphate-buffered saline/0.05 Tween 20) and blocked at room temperature with 1% skimmed milk (Marvel)/PBST for 5 h. Individual wells were washed and incubated with 100 μl of either test sera or a panel of monoclonal antibodies overnight at 4°C, before being washed four times in PBST and incubated for 3 h at room temperature with 100 μl of the respective horseradish peroxidase-conjugated secondary antibody diluted in 1%Marvel/PBST. Wells were washed four times in PBST and incubated at room temperature with 100 μl of substrate development buffer consisting of H_2_O_2_ and o-phenylenediamine dihydrochloride (SigmaFAST). The reaction was stopped with 30μl of 2M H_2_SO_4_ per well and the absorbance read at 492 nm.

### Serum Samples

#### Ethics Statement

This study was carried out in accordance with the recommendations of “the Declaration of Helsinki, and the Kenyan National Scientific and Ethical Review Unit (SERU)” with written informed consent from all subjects. The protocol was approved by SERU, reference number KEMRI/SERU/CGMR-C/001/3139.

#### Kilifi Adults

Samples from life-long adult residents of Junju in Kilifi, Kenya collected in 2008 were used for assay optimization (*n* = 66). These sera were assayed for antibody responses to well-studied recombinant merozoite proteins using monoplex ELISA assays, and the data compared with that generated from the same proteins printed on KILchip v1.0. Five serum samples from adults residing in Sweden who reported no travel to malaria-endemic regions and designated malaria naïve sera (MNS) were used as negative controls. Purified malaria immunoglobulins (MIG) were obtained from a pool of healthy Malawian adults and were used to generate a standard curve as previously described (54). An additional serum pool from Kenyan adult residents of Kilifi, Kenya was designated malaria immune sera (MIS) and served as a second positive control.

### Statistical Analysis

Spot intensities were acquired using the GenePix scanner (Molecular Devices). Background, pre-scan and purification-tag intensities were subtracted (55) before analysis of within sample variability using the Coefficient of Variation (CV)

(1) $$\begin{array}{r} CV=\frac{\sigma }{\mu } \end{array}$$

where σ is the standard deviation and μ the mean fluorescent intensity (MFI).

We used a two-step variance-stabilizing normalization to minimize the systematic variation commonly observed with this type of data. The first step was to handle the differences that could have occurred with different batches of data (machines or day) using the ComBat (SVA package in R). In the second step, variance-stabilizing normalization was used to minimize the systematic variation commonly observed with this type of data (55–58).

## Results

### Recombinant Proteins

**Figure 2:**
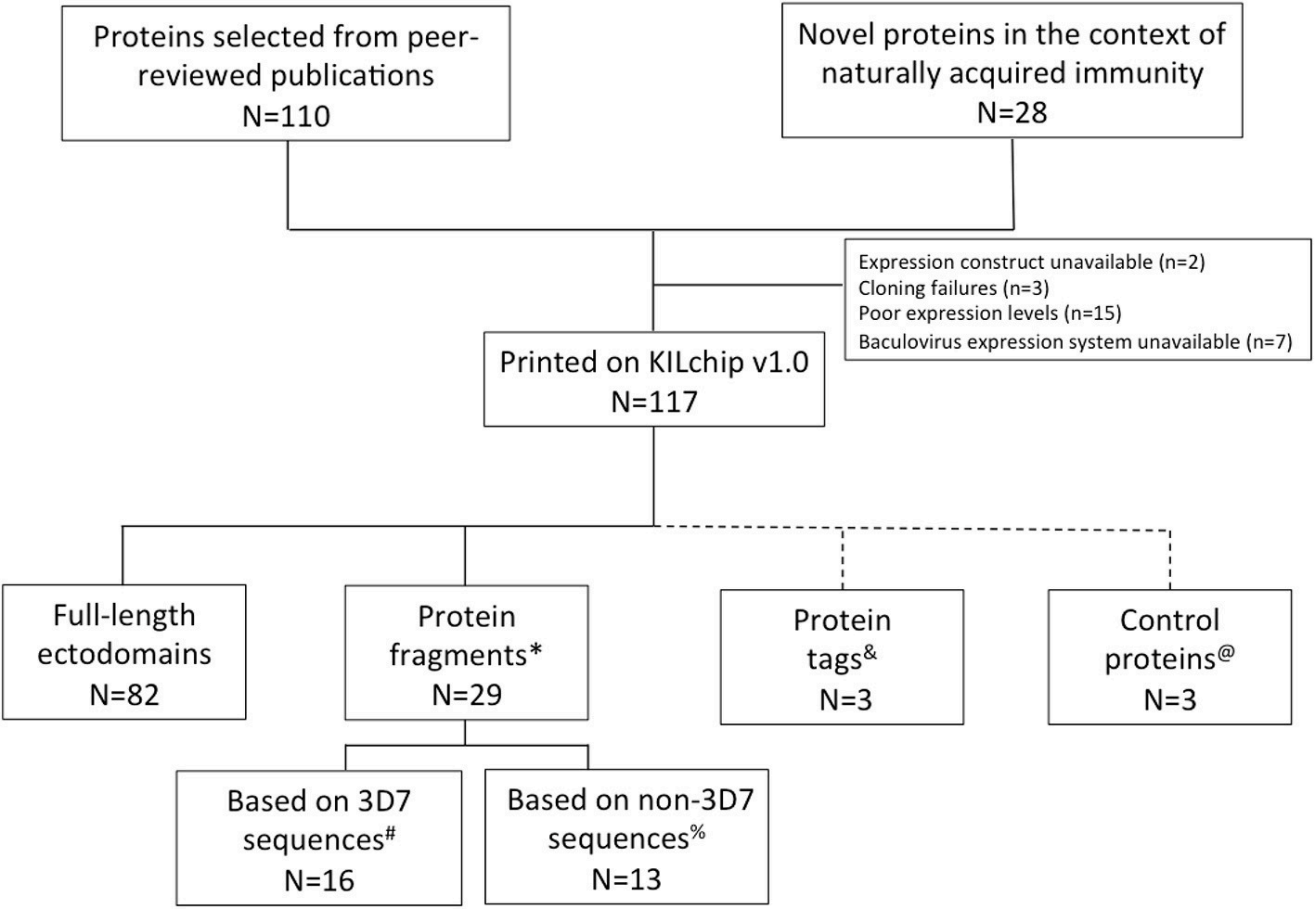
*P. falciparum* merozoite protein panel included in KILchip v1.0. Proteins were either selected from the literature or from a combination of proteomics and bioinformatics analysis. Details of the parasite proteins are provided in Supplementary Table 1. ^*^Protein fragments refers to specific amino acid regions selected from within a full-length protein ectodomain. ^#^The protein fragments based on the 3D7 allele include MSP1-19 (Block 17), MSP1 Block 2 full, MSP1 Block 2 repeat, MSP3, MSPDBL1 N-terminus, MSPDBL1 C-terminus, MSPDBL2 N-terminus, MSPDBL2 C-terminus, 2 extracellular loops of PF3D7_0629500, *Pf* SEA1, SURFIN 4.2 3D7A, SURFIN 4.2 3D7B and the C-terminus of SURFIN 4.2. ^**%**^Thirteen polymorphic variants of the *P. falciparum* merozoite proteins MSP1, MSP2, MSP3, and SURFIN 4.2 were included in KILchip v1.0. These included MSP1 Block 2 from the K1, MAD20, PaloAlto, Wellcome and RO33 alleles, the CH150/9 and DD2 alleles of MSP2, the K1 allele of MSP3 and the K1A and K1B alleles of SURFIN 4.2. ^&^Protein tags refer to specific amino acids or polypeptides fused to target proteins to facilitate their subsequent affinity purification. These include the CD4 hexa-histidine, MBP and GST tags. ^@^Technical controls for the assay included Alexafluor^647^ human IgG, purified human IgG and protein printing buffer.

One hundred and ten *P. falciparum* merozoite proteins were selected from the literature based on their surface localization, known or predicted roles in erythrocyte invasion and associations with protective immunity (32–34, 38–42). Twenty-eight additional novel proteins were identified using a combination of immuno-proteomics and bioinformatics. Of these, 111 *P. falciparum* proteins, 21 of which were novel targets, were successfully printed onto KILchip v1.0 (Figure 2 and Supplementary Table 1). These included 82 full-length ectodomains or the largest predicted extracellular loop of multi-membrane proteins and 29 protein fragments obtained from different regions of eight unique proteins. Thirteen protein fragments corresponded to polymorphic variants of MSP1 (*n* = 7), MSP2 (*n* = 3), MSP3 (*n* = 1) and SURFIN4.2 (*n* = 2) (Figure 2 and Supplementary Table 1). All the remaining proteins were based on the *P. falciparum* 3D7 sequence and in total, 87 unique *P. falciparum* merozoite proteins are printed on KILchip v1.0. Two antigens (MSP2-CH150/9 and PF3D7_0424400) were printed twice to serve as additional internal controls.

### Protein Quality

**Figure 3:**
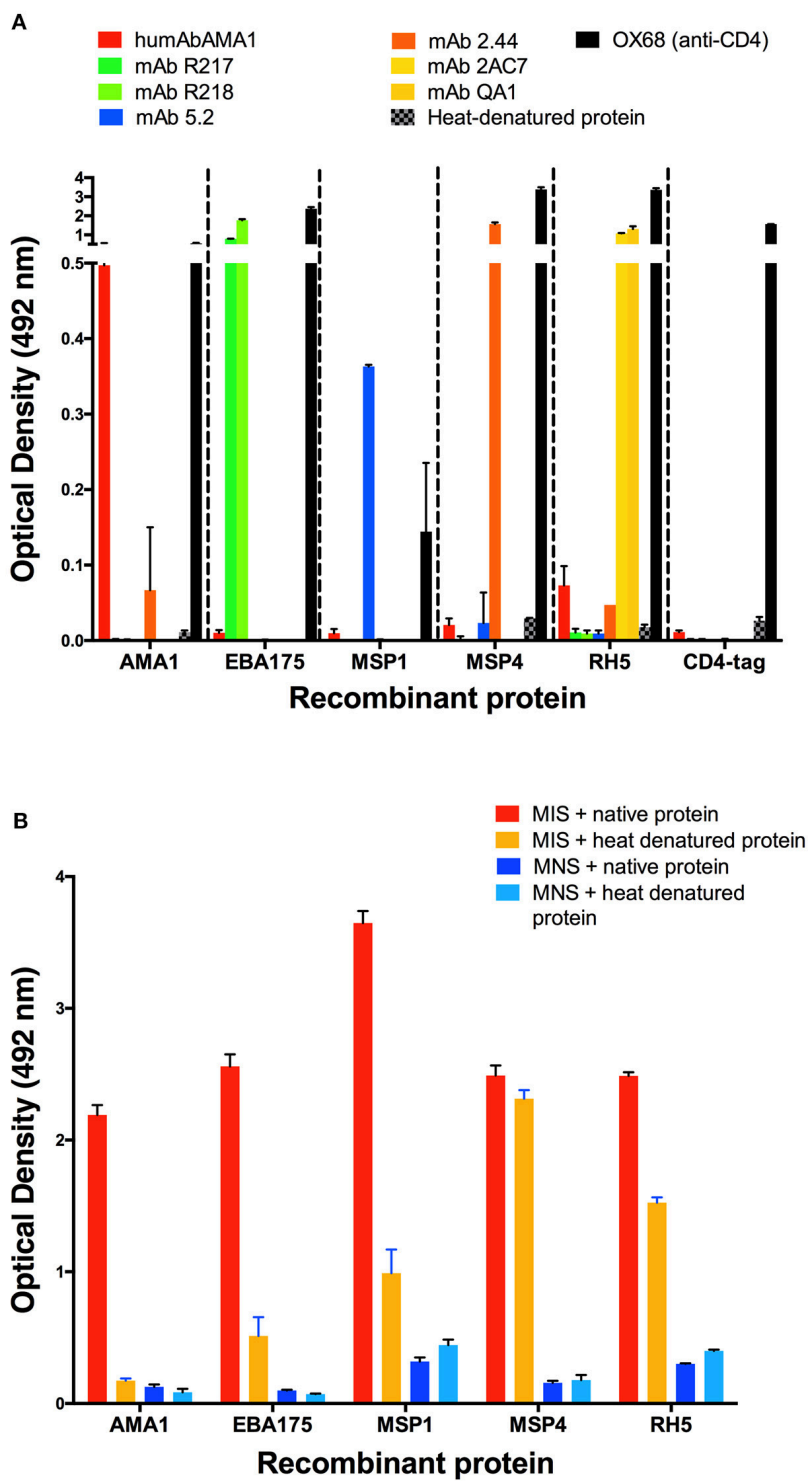
Detection of heat-labile and conformational epitopes in a subset of recombinant proteins. Native and heat-denatured recombinant proteins were tested for reactivity against malaria immune sera (MIS), malaria naïve sera (MNS) and 5 monoclonal antibodies targeting conformational-dependent epitopes. **(A)** The monoclonal antibodies targeting conformational and disulfide-constrained epitopes on AMA1 (humAbAMA1), the F2 domain of EBA175 region II (mAb R217), the F1 domain of EBA175 region II (mAb R218), MSP1 (mAb 5.2), MSP4 (mAb 2.44), RH5 (mAb QA1 and mAb 2AC7) and rat CD4 domain (OX68) were used to measure reactivity against recombinant proteins by ELISA. All recombinant proteins demonstrated high reactivity with their respective monoclonal antibodies only. Low/negligible reactivity was observed after heat-denaturation of the recombinant proteins. **(B)** High antibody reactivity was detected with native recombinant protein. Decreased reactivity was observed with the heat-denatured proteins. Low reactivity was detected with malaria naïve sera (MNS) in both native and heat-denatured proteins.

The quality of the majority of the recombinantly expressed proteins/protein fragments included in KILchip v1.0 have been validated elsewhere, including the demonstration of specific protein-protein interactions (34, 38–41, 43, 45–48). A subset of the well-studied proteins namely: AMA1, EBA175, MSP1, MSP4, and RH5 were evaluated using monoclonal antibodies targeting conformational and disulfide-constrained epitopes in these proteins. Recombinant proteins were tested against humAbAMA1 (59), mAb R217 (60), mAb R218 (60), mAb 2.44 (61), mAb 5.2 (62), mAb 2AC7 (63, 64), and mAb QA1 (64). As shown in Figure 3A, each monoclonal antibody was highly reactive with its respective antigen and showed no reactivity when the target antigen was heat-denatured, confirming the presence of conformational and disulfide-constrained epitopes in the panel of recombinant proteins. As expected, low or negligible reactivity was observed between monoclonal antibodies and off-target recombinant proteins. The presence of heat-labile epitopes was further confirmed by testing native and heat-denatured recombinant proteins for their reactivity with MIS. As shown in Figure 3B, a decrease in immunoreactivity was observed when the proteins were heat-denatured. However, for MSP4, only a minimal drop in immunoreactivity was observed, suggesting the presence of linear epitopes. Collectively, these results suggest that proteins included in KILchip v1.0 were folded correctly and contained heat-labile, conformational epitopes. In addition, circular dichroism analysis of MSP3 and SPATR indicated that these proteins appear to be folded (Supplementary Figure 1).

The novel proteins reported here were expressed using Expi293F cells under the same conditions, lending support to their quality with regards to post-translational modifications and disulfide bond formation. The purity of the novel proteins was assessed using reducing SDS gels and mass-spectrometry analysis. Nineteen (90%) of the novel proteins (*n* = 21) included in KILchip v1.0 were readily detectable on Coomassie stained SDS gels and migrated at a size compatible with their predicted molecular weights (Supplementary Figure 2). Mass-spectrometry analysis confirmed the identity of the majority of recombinant proteins (Supplementary Table 2).

### KILchip v1.0

#### Specificity

**Figure 4:**
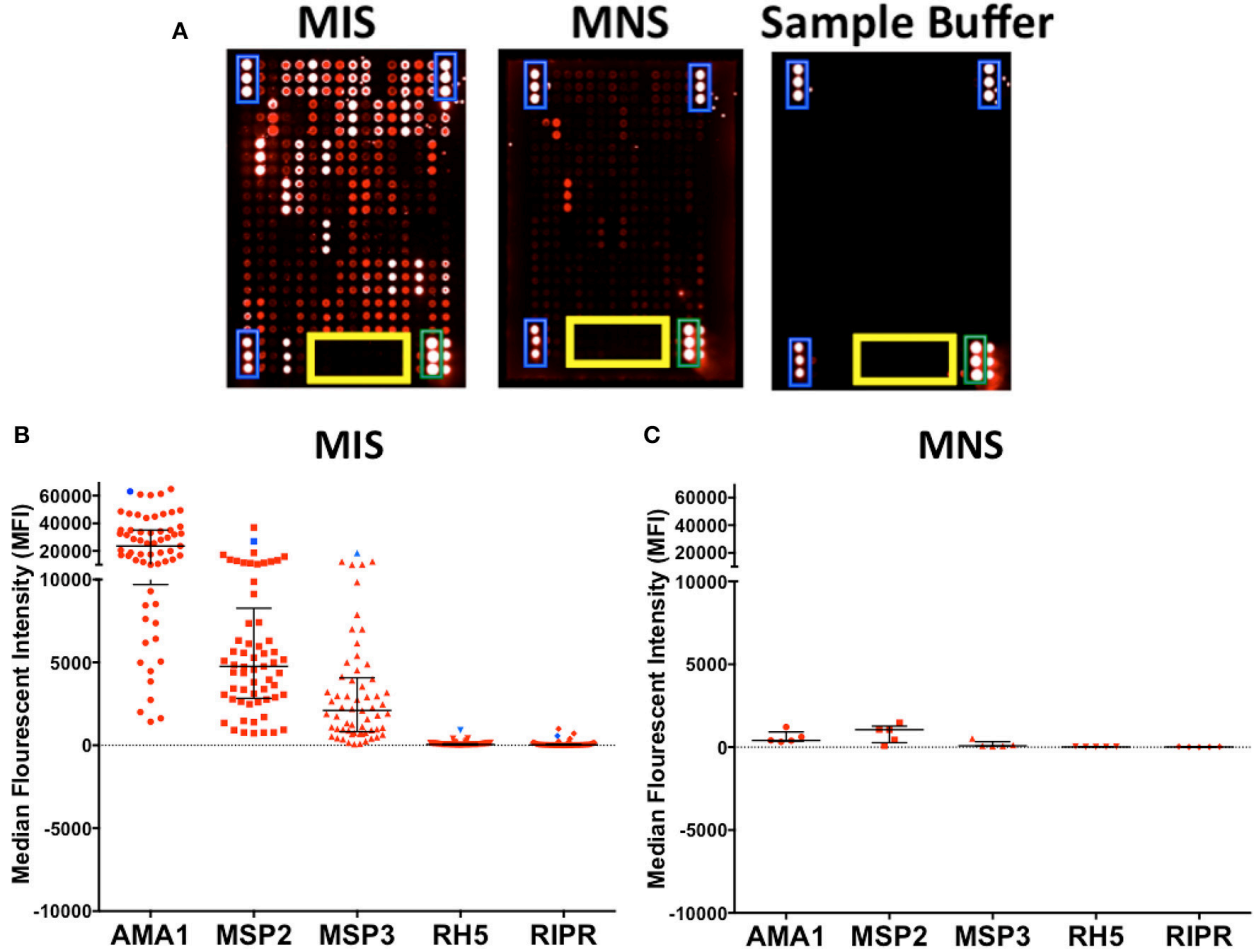
Specificity of KILchip v1.0. Positive and negative controls were used to confirm specificity. **(A)** High antibody reactivity was detected using malaria immune sera (MIS) and low or negligible reactivity was observed with malaria naïve sera (MNS). No reactivity was observed with the sample buffer with the exception of the technical controls. Blue, landmarks; green, commercial human IgG and yellow, printing buffer. **(B)** Antibody reactivity to selected well-characterized merozoite antigens AMA1, MSP2 (3D7), MSP3 (3D7), Rh5, and RIPR varied as expected in MIS (blue) and serum samples from adult residents of Kilifi. Kenya (red). High reactivity to AMA1, MSP2 (3D7), and MSP3 (3D7) and low reactivity to Rh5 and RIPR was observed in the serum samples. **(C)** Antibody reactivity to well-characterized antigens AMA1, MSP2, MSP3, Rh5, and RIPR were all low/negligible when tested in serum samples from Swedish residents.

To confirm the specificity of antibody detection, individual mini-arrays were probed with the following positive and negative controls: (i) malaria immune sera (MIS), (ii) malaria naïve sera (MNS) and (iii) sample buffer consisting of 2% BSA/0.1% Tween 20/HBS. As expected, strong fluorescence was detected on each mini-array for the landmarks (blue) and commercial human IgG (green), whilst none was detected against the printing buffer (yellow) (Figure 4A, all panels). When probed with MIS, high levels of antibody reactivity against multiple *P. falciparum* proteins were clearly visible (Figure 4A, panel 1). In contrast, when probed with MNS, negligible antibody reactivity was observed. Similarly, when probed with sample buffer, nil or minimal reactivity was observed against *P. falciparum* proteins (Figure 4A, panel 2 and 3 respectively).

We further explored the specificity by comparing antibody reactivity to selected, previously characterized proteins in individual samples from Kenyan adults (*n* = 66) and malaria naïve Swedes (*n* = 5). The intensity of antibody reactivity varied between these selected proteins in the order AMA1 > MSP2 > MSP3 > *Pf* RH5 > RIPR (Figure 4B and Supplementary Figure 3). The median (minimum-maximum) fluorescence intensity (MFI) responses to AMA1, MSP2, and MSP3 were 23629 (1426-64840), 4762 (733-37025), and 2105 (72-4069), respectively in the Kenyan adults. MFI responses to RH5 and RIPR were low at 44 (4-914) and 28 (-2-994), respectively (Figure 4B). In contrast, reactivity against all *P. falciparum* proteins was low or negligible when probed with the non-malaria exposed serum from Swedish adults (Figure 4C and Supplementary Figure 3). AMA1, MSP2, and MSP3 are well-characterized immunodominant merozoite antigens while RH5 and RIPR appear to not be primary targets of naturally acquired antibody responses in some studies (32, 33, 65, 66). A direct comparison of antibody responses to multiple antigens can be evaluated using KILchip v1.0 as each protein was printed at the same concentration (250 μg/ml), and would be tested simultaneously with the same sera, providing information on the relative immunogenicity of this panel of merozoite antigens (Supplementary Figure 4).

#### Intra- and Inter-assay Variability

**Figure 5:**
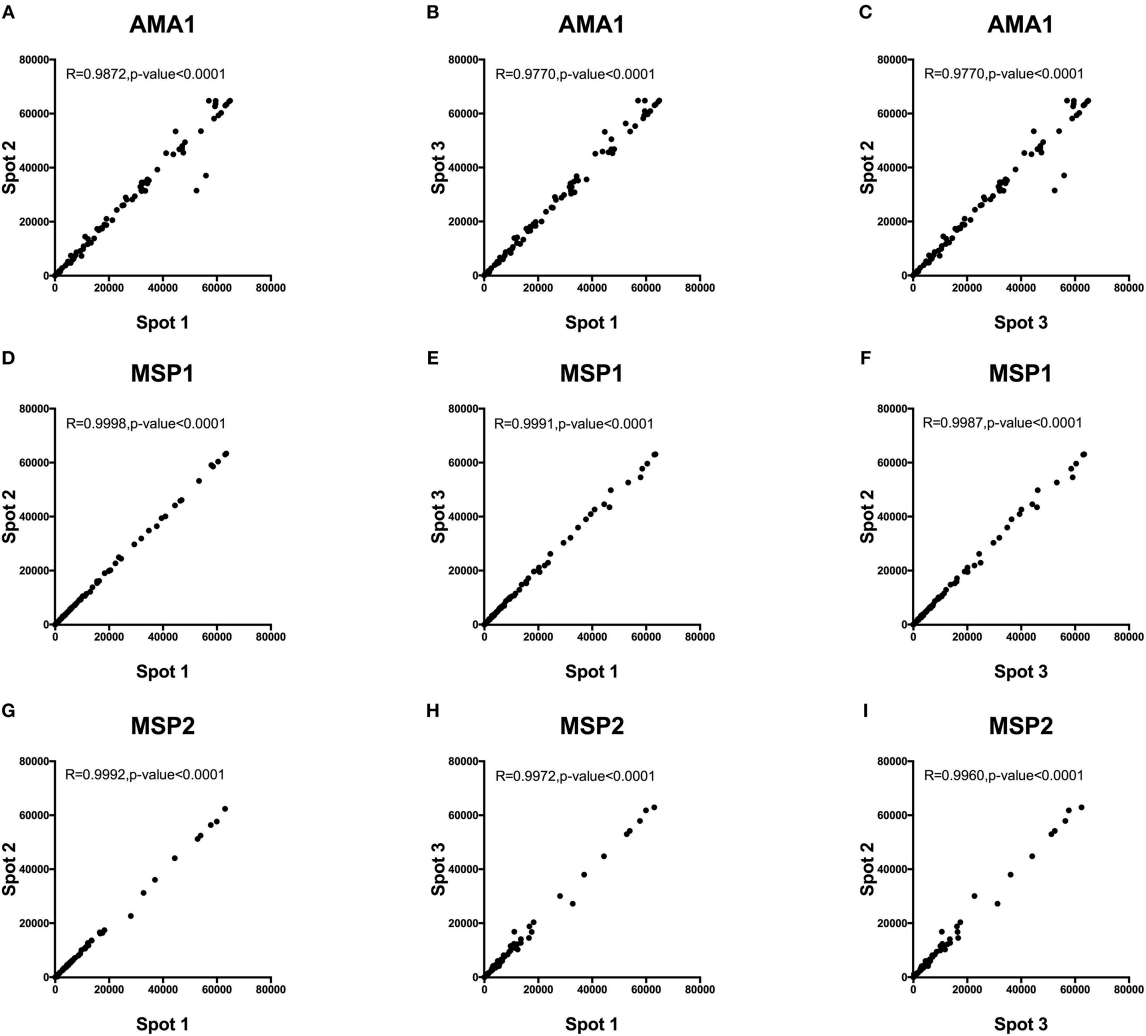
Intra-assay variability of antibody detection by KILchip v1.0. A comparison of antibody responses to triplicate readings of AMA1, MSP1, and MSP2 was measured in 66 serum samples from adults living in the malaria endemic region of Kilifi, Kenya. A strong positive correlation of >0.98 was observed in all three-way scatter plots tested for the three antigens. Correlation between triplicate readings for recombinant AMA1 **(A–C)**, MSP1 **(D–F)** and MSP2 **(G–I)**.

**Figure 6:**
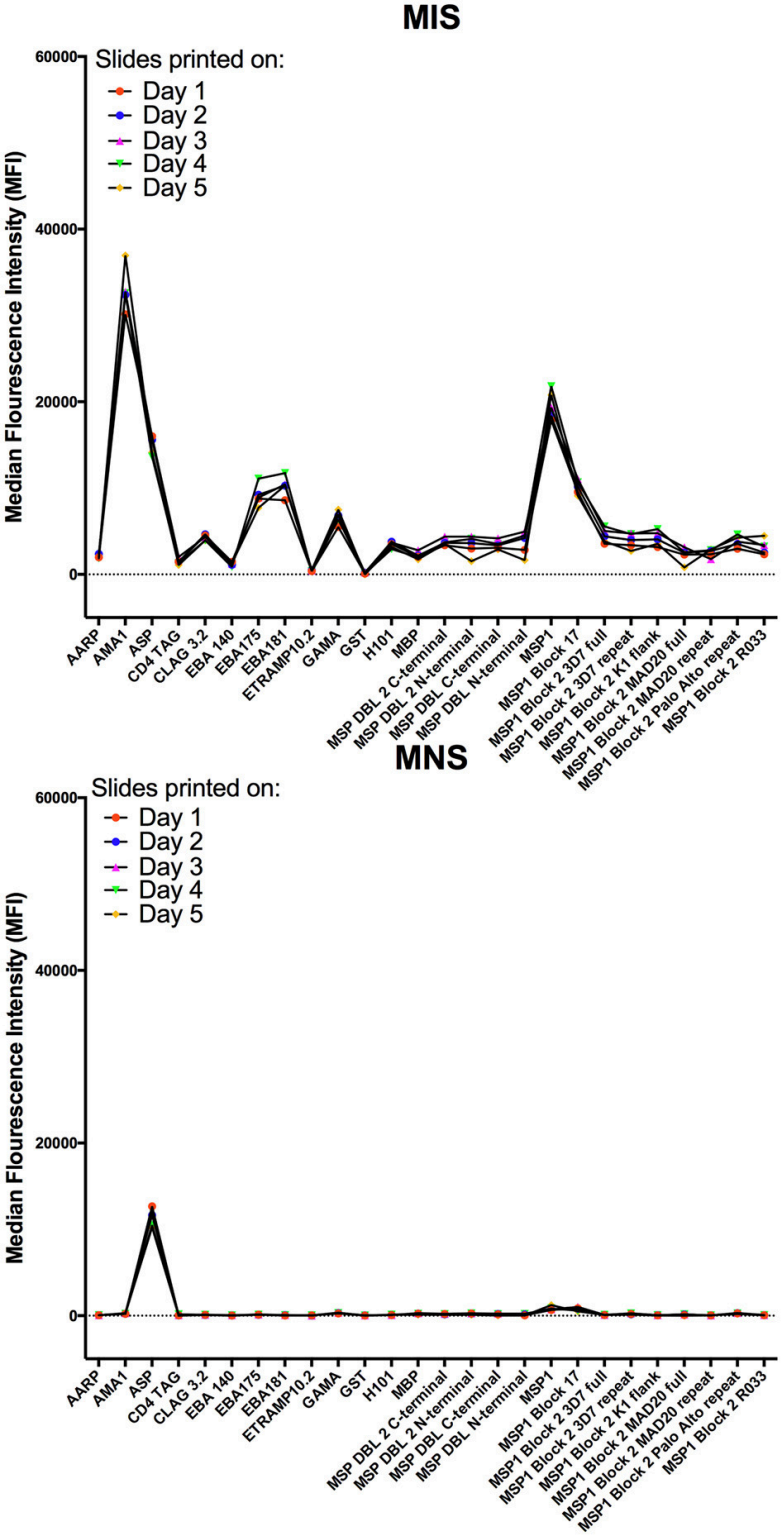
Inter-assay variability of antibody detection by KILchip v1.0. Positive and negative controls were used to measure antibody responses to 5 protein arrays printed on 5 consecutive days using identical printing conditions and the same batch of recombinant proteins. Data presented are the mean MFI values of triplicate readings obtained with MIS (top graph) and MNS (bottom graph) to a subset of the proteins (*n* = 26) printed on each mini-array. Individual proteins are represented as dots and lines have been included to aid visualization.

Intra-assay variability was tested using 66 serum samples obtained from Kenyan adults resident in Kilifi. Antibody measurements between protein spot replicates tested in the same assay and on the same day were strongly positively correlated, Spearman's R > 0.9770, *p* < 0.0001 (Figure 5). Inter-assay variability was tested using the positive and negative control sera. Antibody reactivity was measured repeatedly on five separate microarray slides printed over five consecutive days using identical printing conditions. As shown in Figure 6, the average signal intensities against individual proteins were highly reproducible.

#### Protein Stability on KILchip v1.0

**Figure 7:**
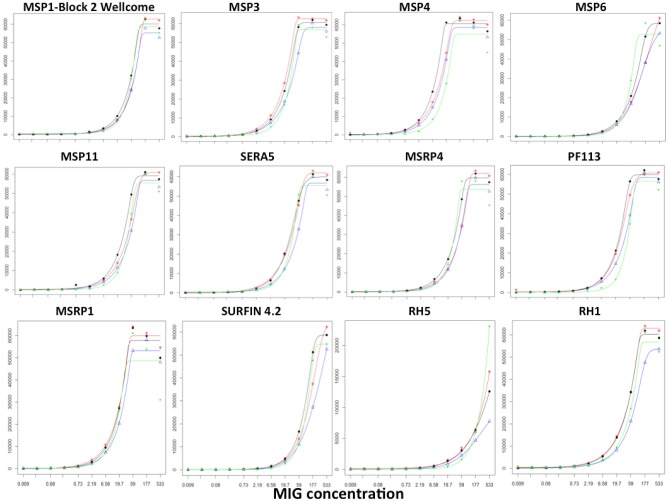
Stability of KILchip v1.0. A reference reagent, MIG, consisting of purified immunoglobulins (98% Total IgG) was used to generate a standard curve by testing a tripling dilution against all antigens printed on the protein array. A batch of slides printed at the same time was tested 24, 40, 54, and 86 days post printing. The standard curve showed good concordance without significant variation. Red, blue, black, and green lines were obtained from measurements obtained on day 24, day 40, day 54, and day 86 post printing of the slides.

To determine the durability of the microarray slides, we measured responses to the same batch of slides over a 3 months period using the reference reagent, MIG. These slides were printed on the same day, with identical printing conditions and the same batch of recombinant proteins. A tripling dilution of MIG was used to measure responses to each protein and to generate a standard curve. We performed a pairwise comparison of the slopes for each of the sigmoidal 5-parameter curves generated for each antigen over the 3-months period (*n* = 4 sigmoidal curves; 6 pairwise comparisons per antigen) (67, 68). We observed no significant differences in the slopes for all 6 pairwise comparisons for 79/111 (71%) proteins (*T*-test; *p* > 0.05). Of the 32 proteins whose slopes differed significantly from each other, eight proteins had a single curve that differed while 24 proteins had 2 or more significantly different slopes. Results from 12/111 (10%) of the proteins printed on the array are shown in Figure 7 and demonstrate that the curves to the majority of proteins were comparable indicating the detection of responses up to 3 months post printing without significant variation.

#### Strong Correlations Between KILchip v1.0 and the Monoplex ELISA

**Figure 8:**
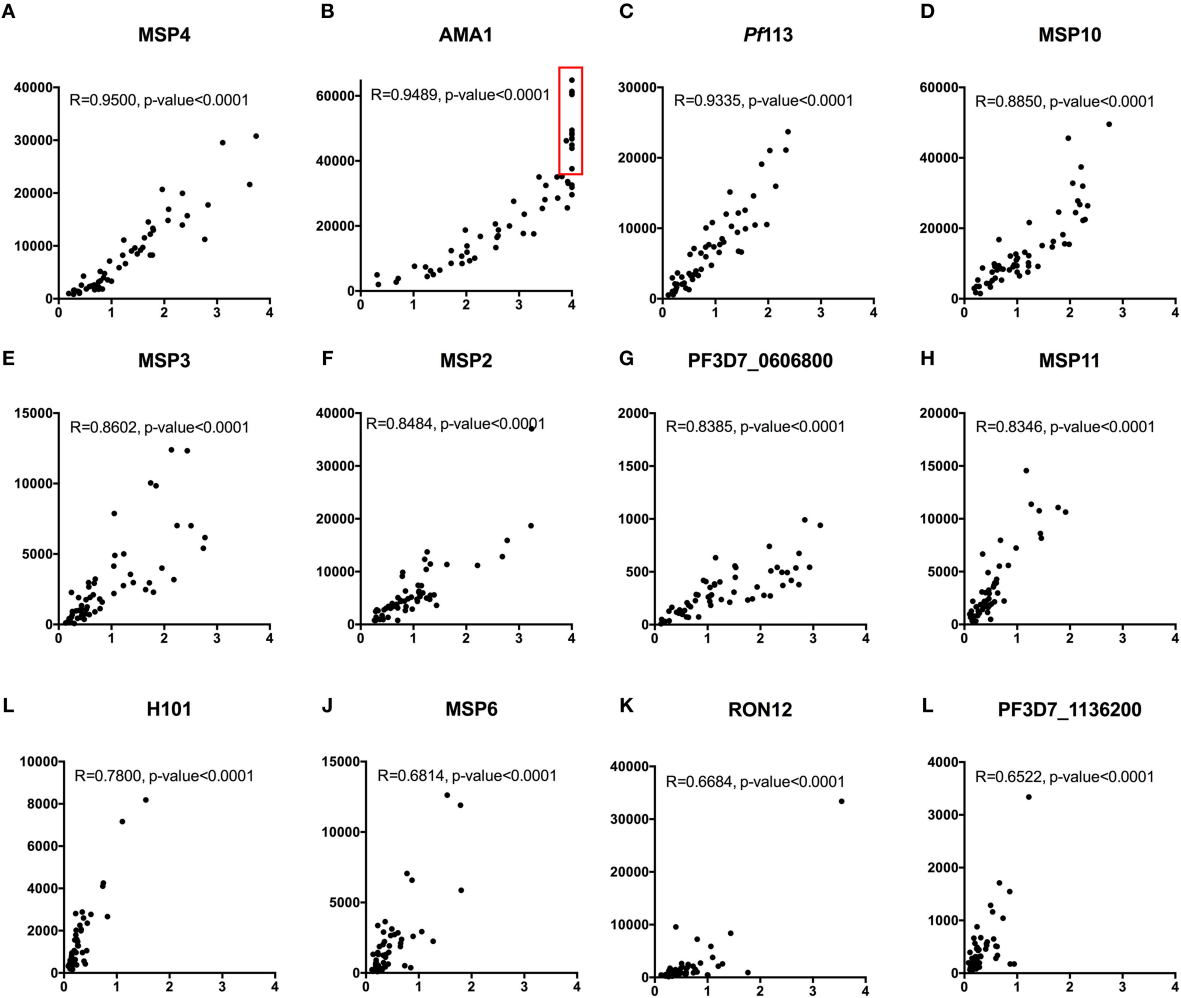
Concordance between KILchip v1.0 and the gold standard ELISA. A comparison of antibody responses to 12 antigens measured simultaneously in KILchip v1.0 and individually by ELISA. Sixty-six serum samples from adults living in the malaria endemic region of Kilifi, Kenya were used to facilitate comparisons. A strong positive correlation of >0.8 was observed in 8 of the antigens **(A–H)** while four antigens **(I–L)** showed a correlation coefficient between 0.65 and 0.78. Red box - serum samples in which antibody responses reached the upper limit of detection when measured by ELISA, yet distinguishable by microarray with MFI values ranging from 40,000 to 60,000.

We compared measurements obtained using 12/111 proteins (representing ~10% of the *P. falciparum* proteins included in the array) printed on KILchip v1.0 vs. the identical proteins using our standardized ELISA in 66 samples from Kenyan adults. As shown in Figures 8A–L, a strong positive Spearman's correlation coefficient R of between 0.65 and 0.95 was observed between antibodies measured by KILchip v1.0 and by the standard ELISA, *P* < 0.0001. Sixty-seven percent of the proteins tested had a correlation coefficient R above 0.8 (Figures 8A–H). A wider dynamic range of antibody measurement was evident in the protein array assay as observed for AMA1 (Figure 8B), where a subset of samples whose MFI values ranged from 40,000 to 60,000 all had an optical density value of 4.0 (red box), the upper limit detectable by ELISA.

## Discussion

KILchip v1.0 is a new protein microarray designed to simultaneously quantify antibodies against multiple *P. falciparum* merozoite proteins and is currently configured to include >100 proteins, the majority of which are full-length ectodomains from secreted and surface exposed proteins. This facilitates the standardized measurement of antibodies against multiple merozoite proteins in cohort studies or in controlled human malaria infections, both of which are currently used to identify and prioritize potential vaccine candidates. It can be utilized to evaluate antibody dynamics and to monitor antibody decay or longevity. The chip is flexible and can be adapted to include fewer or more proteins, allelic variants of selected proteins, full-length proteins or functionally relevant domains of proteins of interest. Adaptations of the chip could expand from the current species-specific focus on *P. falciparum* in KILchip v1.0, and be designed to test additional *Plasmodium* species singly or in parallel. Of particular importance to the research setting in sub-Saharan Africa, the chip is durable, easily stored and transported, and can be shared between partnering laboratories. With modest investments, this tool could transform the pace at which *P. falciparum* antibody response data is generated across the African continent and the same principles could be applied to other infections, ultimately contributing to improvements in health through the development of diagnostics and vaccines.

Although other protein microarrays have been in existence since the early 2,000 s, none has been designed and developed from Africa specifically to study naturally acquired immunity against *P. falciparum* malaria merozoites (13, 15, 69, 70). In addition, KILchip v1.0 consists predominantly of merozoite specific proteins, the majority of which were full-length ectodomains, a considerable improvement from evaluating segments of proteins, as is the case for other pre-existing protein microarrays available for *P. falciparum*. This was enabled by the use of a recently published method for transient protein expression of *P. falciparum* surface proteins in mammalian cells (39). Many of the proteins included in KILchip v1.0 are known to be immunogenic to varying degrees during natural exposure to malaria parasites, but have only been evaluated in a handful of cohort studies (32, 33). Typically, the exact nature, size and quality of antigens tested in these studies vary considerably, making it difficult to accurately compare results (35). Consequently, although some antigens have been studied for nearly 30 years, it is still not clear which proteins should be prioritized as vaccine candidates in clinical trials. KILchip v1.0 now provides a standardized tool that enables head-to-head comparisons of the immunoreactivity of a large number of proteins in multiple cohorts. One such study is already underway in which samples from at least 15 distinct geographical locations spread across 7 African countries have been tested using KILchip v1.0 (SMART: South-South Malaria Antigen Research Partnership) (71) and highlights the potential strength of KILchip v1.0.

We demonstrate that KILchip v1.0 is a sensitive tool for the detection of *P. falciparum* specific antibody responses. It is highly reproducible within and between assays and a strong concordance with the gold-standard monoplex ELISA was observed, comparable to the results reported for the different *P. falciparum* microarrays currently in use (13). Crucially, antibody measurements were stable on KILchip v1.0 up to 3 months post-printing, providing a suitable time frame for the testing of multiple samples. This is probably due to the addition of 60% glycerol to the recombinant proteins at the time of printing which provides a stabilizing effect on proteins preventing degradation (72). Slides are conveniently stored with desiccant at 4°C.

In comparison to our standard ELISA protocol (52, 54), our microarray utilized 0.375 μl of serum to measure responses to all proteins simultaneously, compared to 23 μl required for ELISA assays of the same number of proteins. Similarly, over 100-fold less recombinant protein was required and the laser scanning and data acquisition by the GenePix 4,000 B scanner allowed for much wider dynamic ranges of antibody measurement. Lastly, this is a custom microarray format that could be scaled upwards or downwards and can be adapted to meet specific requirements. In the current version of KILchip v1.0, a single slide has 21 mini-arrays each containing 384 protein spots. This can be re-designed to include more or less proteins, to focus on allelic versions of specific proteins or to facilitate the simultaneous characterization of stage or species-specific antibody responses.

Although we selected 138 proteins/protein fragments for inclusion in KILchip v1.0, we were unable to obtain recombinant protein for 27 targets due to challenges in protein expression. These include targets such as RH4, RAP1-2, RhopH3, GLURP, SERA1, and SERA6 that have also been reported to be difficult to obtain in recombinant form in previous studies (38, 39). Also, the DBL domains of MSPDBL1 and MSPDBL2 have been previously obtained in soluble recombinant form using the baculovirus expression system but this has not yet been established within our laboratories (34). We also observed low antibody responses to some of the merozoite proteins such as RH5 and RIPR, similar to responses measured in adults from Kenya (65) and Mali (66). However, a higher response to these proteins expressed in the wheat germ cell-free expression system has been reported in children in Papua New Guinea (73), suggesting that the protein expression system used may influence the antibody responses measured. Another limitation of the KILchip v1.0 is that the majority of proteins are based on the 3D7 *P. falciparum* isolate which may underestimate responses to highly polymorphic proteins and limit the evaluation of antigenic diversity on humoral responses and immunity. Efforts are underway to generate an additional protein array that will include allelic variants of specific proteins that warrant further study. Lastly, adsorption of proteins onto nitrocellulose-coated slides may interfere with protein structure and consequently with the detection of conformation-dependent antibody responses (74). However, these and other solid-surface platforms such as in ELISAs are widely utilized for antibody detection for vaccine candidate discovery and prioritization in multiple infectious and non-infectious diseases.

Current and future versions of KILchip v1.0 will be essential to multi-center prospective cohort studies designed to identify correlates of protection, allowing the research community to rapidly compare results head-to-head, and fast track the prioritization of new and old potential vaccine candidates. This would bridge an important gap for the urgently needed evidence base that could guide the development of the next generation of malaria vaccines. Efforts are underway to make this a resource that could be provided at a minimal cost to the malaria research community.

## Author Contributions

FO, JR, and KevM conceived the study. GK and JT designed the array. GK, JT, RK, MC, SH, JN, CK, MR, RF, RY, EC, TC, JM, and FG performed the experiments, GK wrote the paper with contributions from JT, KenM, NK, and FO. SK, LM, and PB provided helpful discussions. AF, KT, JB, and DC provided reagents for the array design and testing. All authors read and approved the final manuscript.

### Conflict of Interest Statement

The authors declare that the research was conducted in the absence of any commercial or financial relationships that could be construed as a potential conflict of interest.

## Supplementary Material

Supplementary Table 1 List of P. falciparum merozoite proteins included in KILchip v1.0. The details of the P. falciparum merozoite proteins provided include the expression system used, the region of the protein expressed, the P. falciparum strain the protein was based on and the recombinant expression levels achieved. The expression levels were categorized as low, intermediate and high based on the concentration of purified protein obtained following transfection of a standard concentration of 50.0 × 10^6^ Expi293F cells. The protein concentration following purification and concentration of low, intermediate and high expressors were < 100 μg/ml, 100–250 μg/ml, and >250 μg/ml, respectively. The expression levels are given as guide only given the variability that may be observed with different expression batches. We observed no differences in the background signals when malaria naïve sera were tested against recombinant proteins grouped according to their expression levels (Kruskal-Wallis H Test for trend: *p*-value = 0.5975) (data not shown).

Supplementary Table 2 Mass-spectrometry confirmation of novel Plasmodium falciparum recombinant proteins. Mass-spectrometry confirmation of the novel Plasmodium falciparum recombinant proteins included in KILchip v1.0. Data for PF3D7_0830500, PF3D7_1025300, PF3D7_1229300, PF3D7_1252300, PF3D7_1237900, PF3D7_1343700, and PF3D7_0629500_SEG2 are not yet available.

Supplementary Figure 1 Circular dichroism spectroscopy on recombinant SPATR and MSP3. Circular Dichroism (CD) spectra graphs for SPATR (A) and MSP3 (B) proteins are plotted from mean residual ellipticity [θ] (mDeg · cm2·dmol−1·res−1) as a function of the wavelength, λ (nM). The ellipticity for the blank (phosphate buffer (50mM NaH2PO4 pH 8.0, 0.1M NaCl)) was subtracted from the ellipticity for the protein samples before calculation of the mean residue ellipticity [θ].

Supplementary Figure 2 Purity of novel Plasmodium falciparum proteins. Coomassie stained SDS gels showing 19 purified recombinant proteins. The numbers in bracket indicate the predicted molecular masses (in KDa) for each protein. Protein bands for PF3D7_1343700 and PF3D7_0629500_SEG2 were not readily visible on a coomassie stained SDS gel. Protein bands for AMA1 and CD4-hexa-histidine tags were included as controls.

Supplementary Figure 3 Antibody responses to previously characterized merozoite proteins. The protein spots corresponding to previously characterized merozoite antigens AMA1, MSP2 (3D7), MSP3 (3D7), Rh5, and RIPR are shown in protein arrays probed with MIS and MNS sera. Red-AMA1, Yellow-MSP2, Blue-MSP3, Orange-RH5, and Green-RIPR.

Supplementary Figure 4 Immunoreactivity of all the recombinant proteins printed on KILchip v1.0 measured using a pool of sera from naturally exposed adults. The immunoreactivity of the 111 novel proteins was tested using malaria immune sera (MIS, red bars) and malaria naïve sera (MNS, blue bars). Bar charts show mean plus standard deviation; *n* = 2.
