# Supplementary material for: KILchip v1.0: A Novel Plasmodium falciparum Merozoite Protein Microarray to Facilitate Malaria Vaccine Candidate Prioritization
Source: Front Immunol. 2018 Dec 11;9:2866. doi: 10.3389/fimmu.2018.02866 (PMC6298441; doi:10.3389/fimmu.2018.02866)
Supplement: Supplementary Table 2 — Mass-spectrometry confirmation of novel Plasmodium falciparum recombinant proteins. Mass-spectrometry confirmation of the novel Plasmodium falciparum recombinant proteins included in KILchip v1.0. Data for PF3D7_0830500, PF3D7_1025300, PF3D7_1229300, PF3D7_1252300, PF3D7_1237900, PF3D7_1343700, and PF3D7_0629500_SEG2 are not yet available. [file Table_2.DOCX]

| **Accession** | **Description** | **Score** | **Coverage** | **# Unique peptides** | **#PSMs** | **Mw (kDa)** | **Calc. pI** |
| --- | --- | --- | --- | --- | --- | --- | --- |
| AMA1 | organism=Plasmodium_falciparum_3D7 \| product=apical membrane antigen 1 (AMA1) \| location=Pf3D7_11_v3:1293856-1295724(+) \| length=622 \| sequence_SO=chromosome \| SO=protein_coding | 6009.30 | 41.64 | 37 | 403 | 72.0 | 5.54 |
| PF3D7_073800.2 | organism=Plasmodium_falciparum_3D7 \| product=Plasmodium exported protein, unknown function \| location=Pf3D7_07_v3:1321306-1322367(+) \| length=293 \| sequence_SO=chromosome \| SO=protein_coding | 3383.72 | 51.88 | 16 | 313 | 32.1 | 5.03 |
| PF3D7_0206200 | organism=Plasmodium_falciparum_3D7 \| product=metabolite/drug transporter, putative \| location=Pf3D7_02_v3:249030-250727(+) \| length=565 \| sequence_SO=chromosome \| SO=protein_coding | 1955.88 | 3.36 | 3 | 54 | 62.6 | 7.05 |
| PF3D7_1462300 | organism=Plasmodium_falciparum_3D7 \| product=conserved Plasmodium protein, unknown function \| location=Pf3D7_14_v3:2524921-2528994(-) \| length=1357 \| sequence_SO=chromosome \| SO=protein_coding | 5079.21 | 25.35 | 37 | 322 | 161.1 | 6.42 |
| PF3D7_1401600 | organism=Plasmodium_falciparum_3D7 \| product=Plasmodium exported protein (PHISTb), unknown function \| location=Pf3D7_14_v3:61364-62988(-) \| length=478 \| sequence_SO=chromosome \| SO=protein_coding | 5664.57 | 46.44 | 20 | 455 | 55.8 | 6.58 |
| PF3D7_1460600 | organism=Plasmodium_falciparum_3D7 \| product=inner membrane complex protein, putative \| location=Pf3D7_14_v3:2473758-2474204(+) \| length=148 \| sequence_SO=chromosome \| SO=protein_coding | 3741.25 | 70.95 | 11 | 235 | 17.1 | 6.14 |
| PF3D7_0831400 | organism=Plasmodium_falciparum_3D7 \| product=Plasmodium exported protein, unknown function \| location=Pf3D7_08_v3:1344405-1345487(-) \| length=302 \| sequence_SO=chromosome \| SO=protein_coding | 1567.17 | 29.47 | 7 | 88 | 36.4 | 6.09 |
| PF3D7_1455300 | organism=Plasmodium_falciparum_3D7 \| product=conserved Plasmodium protein, unknown function \| location=Pf3D7_14_v3:2259465-2261297(+) \| length=610 \| sequence_SO=chromosome \| SO=protein_coding | 3347.39 | 35.25 | 13 | 324 | 69.5 | 9.20 |
| PF3D7_0925900 | organism=Plasmodium_falciparum_3D7 \| product=conserved Plasmodium protein, unknown function \| location=Pf3D7_09_v3:1040713-1041516(+) \| length=217 \| sequence_SO=chromosome \| SO=protein_coding | 2644.62 | 41.47 | 11 | 254 | 24.7 | 5.63 |
| PF3D7_1407800 | organism=Plasmodium_falciparum_3D7 \| product=plasmepsin IV (PM4) \| location=Pf3D7_14_v3:283086-284435(+) \| length=449 \| sequence_SO=chromosome \| SO=protein_coding | 5894.32 | 50.56 | 20 | 318 | 51.0 | 5.54 |
| PF3D7_1345100 | organism=Plasmodium_falciparum_3D7 \| product=thioredoxin 2 (TRX2) \| location=Pf3D7_13_v3:1809736-1810521(-) \| length=157 \| sequence_SO=chromosome \| SO=protein_coding | 3172.13 | 50.96 | 12 | 147 | 18.6 | 9.76 |
| PF3D7_1105800 | organism=Plasmodium_falciparum_3D7 \| product=conserved Plasmodium protein, unknown function \| location=Pf3D7_11_v3:250918-252872(+) \| length=266 \| sequence_SO=chromosome \| SO=protein_coding | 274.48 | 33.46 | 3 | 46 | 30.6 | 5.12 |
| PF3D7_1137300 | organism=Plasmodium_falciparum_3D7 \| product=conserved Plasmodium membrane protein, unknown function \| location=Pf3D7_11_v3:1466197-1468266(-) \| length=689 \| sequence_SO=chromosome \| SO=protein_coding | 309.38 | 3.34 | 2 | 12 | 81.6 | 8.97 |
| PF3D7_0525800 | organism=Plasmodium_falciparum_3D7 \| product=membrane skeletal protein IMC1-related \| location=Pf3D7_05_v3:1071247-1072149(+) \| length=300 \| sequence_SO=chromosome \| SO=protein_coding | 502.67 | 35.67 | 5 | 25 | 34.3 | 6.98 |
| PF3D7_0629500_SEG1 | organism=Plasmodium_falciparum_3D7 \| product=amino acid transporter, putative \| location=Pf3D7_06_v3:1213948-1216005(-) \| length=606 \| sequence_SO=chromosome \| SO=protein_coding | 767.77 | 8.25 | 3 | 63 | 68.8 | 9.61 |
