# Supplementary figures and images for: KILchip v1.0: A Novel Plasmodium falciparum Merozoite Protein Microarray to Facilitate Malaria Vaccine Candidate Prioritization
Source: Front Immunol. 2018 Dec 11;9:2866. doi: 10.3389/fimmu.2018.02866 (PMC6298441; doi:10.3389/fimmu.2018.02866)

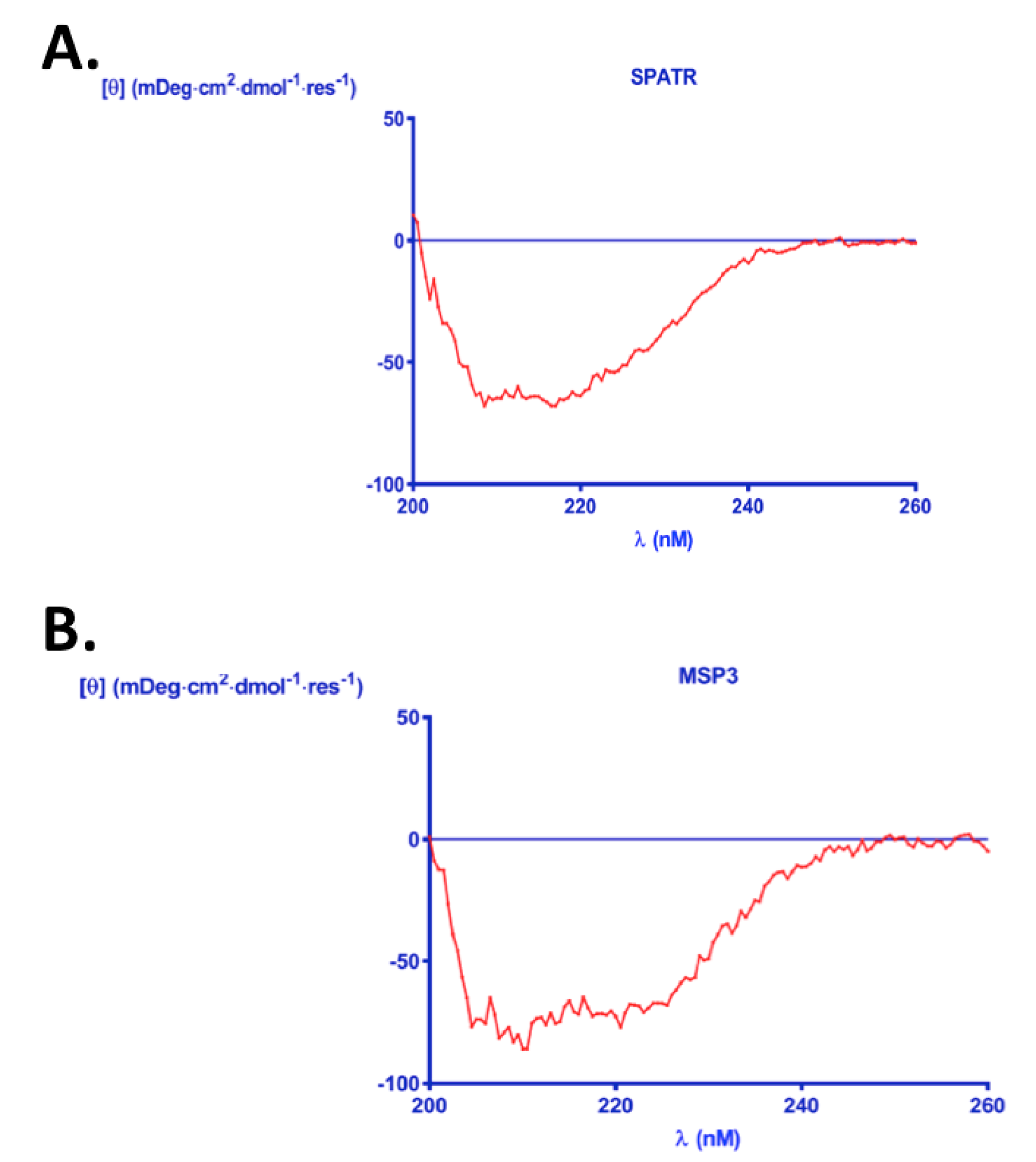

Supplement: Supplementary Figure 1 — Circular dichroism spectroscopy on recombinant SPATR and MSP3. Circular Dichroism (CD) spectra graphs for SPATR (A) and MSP3 (B) proteins are plotted from mean residual ellipticity [θ] (mDeg · cm2·dmol−1·res−1) as a function of the wavelength, λ (nM). The ellipticity for the blank (phosphate buffer (50mM NaH2PO4 pH 8.0, 0.1M NaCl)) was subtracted from the ellipticity for the protein samples before calculation of the mean residue ellipticity [θ]. [file Image_1.TIFF]

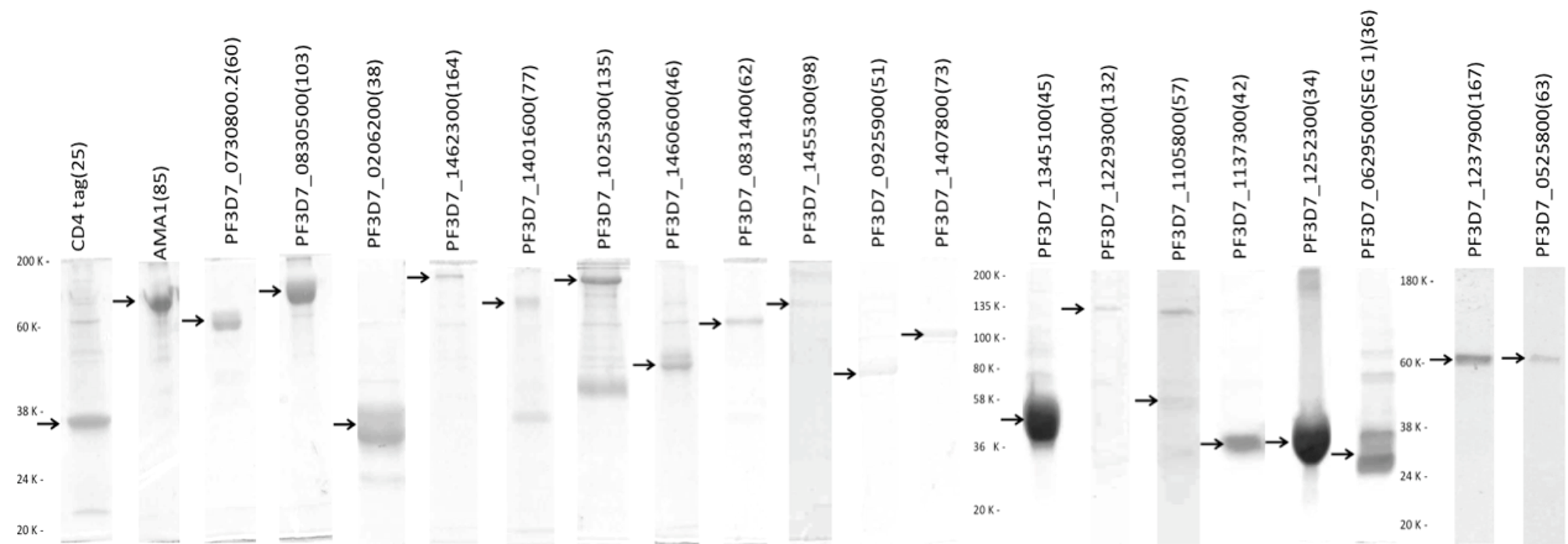

Supplement: Supplementary Figure 2 — Purity of novel Plasmodium falciparum proteins. Coomassie stained SDS gels showing 19 purified recombinant proteins. The numbers in bracket indicate the predicted molecular masses (in KDa) for each protein. Protein bands for PF3D7_1343700 and PF3D7_0629500_SEG2 were not readily visible on a coomassie stained SDS gel. Protein bands for AMA1 and CD4-hexa-histidine tags were included as controls. [file Image_2.TIFF]

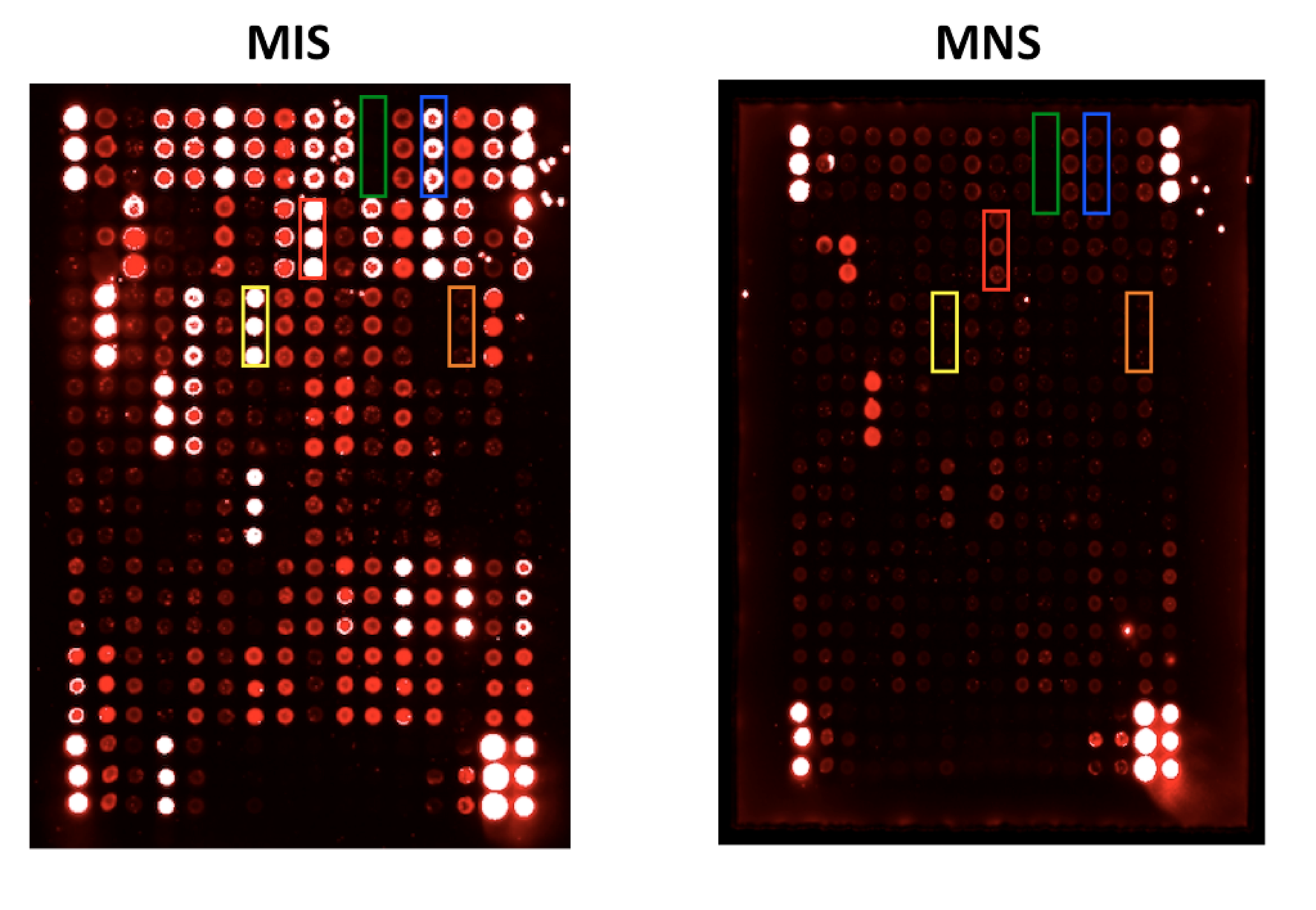

Supplement: Supplementary Figure 3 — Antibody responses to previously characterized merozoite proteins. The protein spots corresponding to previously characterized merozoite antigens AMA1, MSP2 (3D7), MSP3 (3D7), Rh5, and RIPR are shown in protein arrays probed with MIS and MNS sera. Red-AMA1, Yellow-MSP2, Blue-MSP3, Orange-RH5, and Green-RIPR. [file Image_3.TIFF]

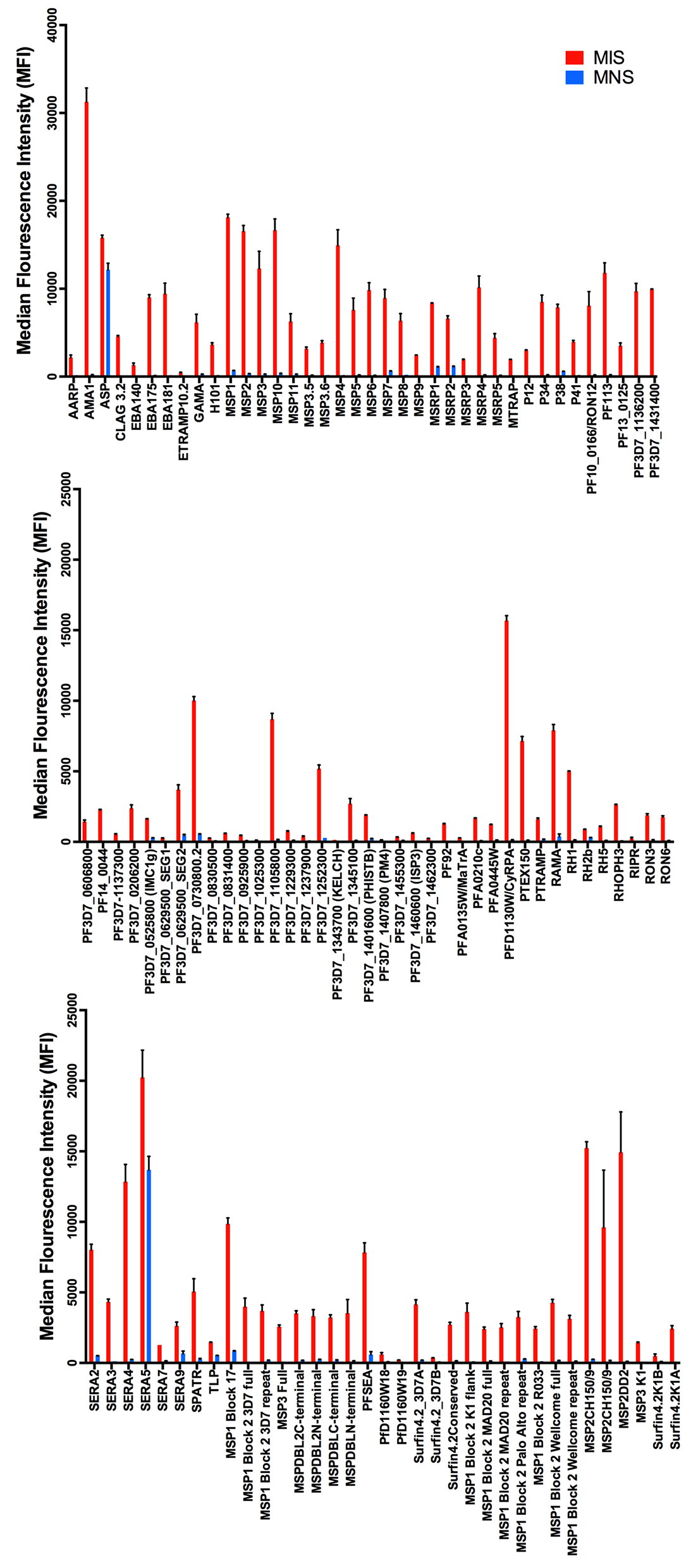

Supplement: Supplementary Figure 4 — Immunoreactivity of all the recombinant proteins printed on KILchip v1.0 measured using a pool of sera from naturally exposed adults. The immunoreactivity of the 111 novel proteins was tested using malaria immune sera (MIS, red bars) and malaria naïve sera (MNS, blue bars). Bar charts show mean plus standard deviation; n = 2. [file Image_4.TIFF]
